# Linking Self- and Other-Focused Emotion Regulation Abilities and Occupational Commitment among Pre-Service Teachers: Testing the Mediating Role of Study Engagement

**DOI:** 10.3390/ijerph18105434

**Published:** 2021-05-19

**Authors:** Sergio Mérida-López, Natalio Extremera, Maria José Chambel

**Affiliations:** 1 Department of Social Psychology, Social Work, Social Anthropology and East Asian Studies, University of Malaga, 29071 Malaga, Spain; nextremera@uma.es; 2 CICPSI, Faculdade de Psicologia, Universidade de Lisboa, 2649-013 Lisboa, Portugal; mjchambel@psicologia.ulisboa.pt

**Keywords:** emotion regulation abilities, study engagement, occupational commitment, pre-service teachers, mediation

## Abstract

This investigation aimed to explore the mediator role of study engagement (i.e., study vigor and dedication) in the association between self- and other-focused emotion regulation abilities and occupational commitment in a sample of pre-service teachers. The sample was comprised of 249 students (65.5% female; Mage = 27 years) of a master’s degree in teacher training for secondary education. Results showed the relationship between self-focused emotion regulation ability and occupational commitment to be fully mediated by levels of vigor. No significant results were found regarding a mediator model involving other-focused emotion regulation as predictor. Although these findings warrant prospective replication, they provide evidence that development of self-focused emotion regulation skills (rather than other-focused skills) may facilitate occupational commitment among beginning teachers through desirable states that facilitate energy and reduce the likelihood of fatigue at work. These results are discussed in terms of their practical implications for developing interventions to improve pre-service teachers’ well-being and commitment.

## 1. Introduction

In recent decades, a number of studies have reported that teachers face a wide variety of demands in their daily working life that lead many of them to feel exhausted, disengaged, and dissatisfied [1]. For instance, teachers often perceive their work environment as threatening with contextual factors such as pupil misbehavior, lack of organizational support, and emotionally demanding situations being commonly encountered [1,2,3]. The high prevalence of these contextual demands leading to increased stress and exhaustion calls for the exploration of the protective factors that may reduce the impact of such contextual variables on public health and retention issues [1]. Teacher-related demands and educator stress affects not only in-service but also pre-service teachers, that is, individuals in the transition from university into the professional world, who may fail to match the required tasks and demands related to teaching practice [2,3,4]. Entering the teaching profession and experiencing the common shock of reality regarding teacher-related demands may cause a variety of negative emotions that may erode their attitudes toward their career [2,3]. In this regard, issues regarding reduced commitment and increasing intention to quit among beginning and newly-qualified teachers merit serious attention as they entail issues of an economic, educational, and social nature [5,6]. However, individuals do show marked differences in their responses to affective events and stressors [7].

Past research has demonstrated that several individual factors may contribute to explaining why some teachers feel drained by their work whereas others under the same organizational conditions feel engaged with their vocation [8]. Similarly, individual differences regarding availability of personal resources among pre-service and beginning teachers contribute to explaining, to some extent, how effective they are at managing stress-eliciting events which, in turn, may determine subsequent well-being and distal outcomes such as commitment [2,9,10]. Therefore, pre-service teachers’ personal resources are increasingly considered as key factors for professional efficacy and retention [11,12]. The existing literature on teachers’ personal resources underlines the importance of increasing efforts for exploring and promoting these positive dimensions among in-service and pre-service teachers to ensure a sustainable development of health and well-being among educators, students, and educational communities [1,8,10].

It is well documented that some difficulties in managing negative emotions associated with teaching practice are linked to negative work attitudes such as reduced commitment, dissatisfaction, and increased burnout and intention to quit [2,8]. However, less is known about the role that personal resources may play in the prediction of positive occupational attitudes and outcomes among individuals in the transition from university into the teaching profession [13]. There is research advocating that primary preventive efforts are needed to sustain a healthier and more committed teaching workforce [1,11]. On this basis, the current work aims to provide novel empirical evidence on the associations among personal resources relating to emotion regulation, engagement, and occupational commitment among pre-service teachers. 

**Figure 1:**
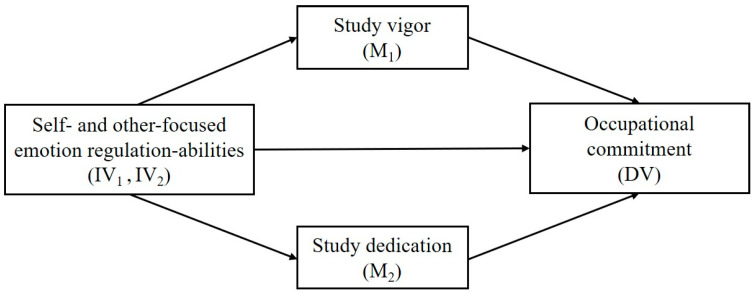
The proposed conceptual model.

This study examines the potential mediating role of vigor and dedication in the relationship between self- and other-focused emotion regulation abilities and occupational commitment, using objective and self-report measures in a sample of future secondary-school teachers. In doing so, several contributions to current knowledge on emotional intelligence (EI) among future teachers are made. First, this work applies the job demands-resources theory to the pre-occupational context examining the predictive role of self- and other-focused emotion regulation abilities in relevant motivational (i.e., study engagement) and attitudinal (i.e., occupational commitment) criteria (see Figure 1). There is extensive evidence on the protective role of EI dimensions regarding teacher burnout [8,14]. However, the literature on the role of EI abilities as predictors of teaching-related commitment, a key variable predicting efficacy and retention, is limited. Thus, the current study may add to a burgeoning body of research aiming at increasing knowledge regarding the emotional abilities that may be trained among beginning teachers not only to reduce work stress and burnout but also to increase positive job attitudes such as occupational commitment [1,11].

A second contribution of the current work relates to the assessment of emotion regulation with a performance-based EI measure as opposed to using self-report instruments and so it may add to limited knowledge with objective EI measures [14,15]. Third, this research may provide a more fine-grained understanding on the associations among self- and other-focused emotion regulation abilities, study engagement, and commitment than previously available. Fourth, self- and other-focused emotion regulation abilities are explored as individual factors expected to facilitate engagement and commitment among individuals between the two realms of teacher training and professional experience in secondary education. On the one hand, this research may help to delineate the mechanisms through which a more engaged and committed teaching forced is retained [10]. On the other hand, findings from this study may be relevant for designing EI training in the intersection between the educational and the occupational domains so well-being and commitment are facilitated [7].

## 2. Theoretical Background and Study Hypotheses

### 2.1. Occupational Commitment as a Critical Dimension in the Teaching Context

Occupational commitment has been defined as the strength of motivation to work in a chosen career role [16]. Further, Hackett, et al. [17] defined occupational commitment as “the level of attachment to, or desire to work in, a particular career role.” Scholars have devoted growing efforts to examining the correlates of teachers’ occupational commitment and there is consistent evidence showing the effects of this attitudinal dimension on teacher performance, work involvement, and intention to quit [18]. Further, pre-service teachers’ occupational commitment is predictive of their withdrawal intentions [12,18]. An alarming number of beginning teachers are leaving their career in their first years of teaching experience [2,5]. This major concern posits a challenge for administrators, scholars, and practitioners as individuals trained for the teaching workforce are increasingly leaving their career for other job opportunities [12,18,19]. Therefore, identifying the individual factors related to commitment among future teachers is critical to understand how occupational well-being and retention could be promoted through practical efforts [2].

Teaching work involves high emotional demands and emotional work, where numerous affective events and situations with students, families, and colleagues take place, and hence, emotional management seems highly determinant of well-being and commitment in this occupational context [1,20]. In this regard, a dimension related to emotional information processing, namely EI, is increasingly considered as a key aspect for improving desirable outcomes among pre-service teachers [11].

### 2.2. Emotion Regulation Abilities as Predictors of Occupational Commitment

Following the ability model of EI [21], this construct is defined as the “the ability to perceive accurately, appraise, and express emotion; the ability to access and/or generate feelings when they facilitate thought; the ability to understand emotion and emotional knowledge; and the ability to regulate emotions to promote emotional and intellectual growth.” Accordingly, EI includes four interrelated abilities: perceiving emotions, using emotions, understanding emotions, and regulating emotions in oneself and others [21].

There is consistent evidence on the positive effects of EI abilities on occupational well-being and commitment among both in-service and pre-service teachers [11,20]. Nonetheless, emotion regulation has been underscored as the most salient EI ability given its consistent positive effects on occupational well-being, satisfaction, and performance in emotionally demanding contexts including teaching [1,22,23]. The emotion regulation ability (ERA) dimension of EI is defined as the ability to effectively manage one’s own (or others’) emotions to achieve a desired outcome, including skills such as being able to modify an emotional response or evaluating the appropriateness of certain emotions depending on the context [15]. According to the ability approach, ERA is generally measured with situational judgment tests indicating an individual’s capacity to implement a given emotion regulation strategy in emotional situations [15]. However, there is limited evidence on these measures in comparison with self-report instruments [9,24]. Although this can be understood considering self-report EI measures are short and easy to administer, additional studies using performance-based EI tests are needed to provide a deeper understanding on the effects of adaptive emotional management processes among pre-service teachers.

With regard to the conceptualization of ERA, self-focused emotion regulation ability is referred to as the capacity to implement effective actions for changing or maintaining desirable emotional states in oneself, whereas other-focused emotion regulation ability relates to the implementation of effective actions to achieve a specific emotional outcome in social interactions involving other people [15,25]. While self-focused emotion regulation abilities may protect pre-service teachers from the deleterious impact of contextual factors (e.g., academic demands) on their health, well-being, and motivation, other-focused emotion regulation abilities are relevant for facilitating social interactions with others [26]. On this theoretical basis, EI dimensions including emotion regulation are found to facilitate a more adaptive functioning in higher education [7,27]. However, existing studies have been mainly focused on emotion regulation as a whole and the motivational and attitudinal correlates of self- and other-focused emotion regulation abilities have not yet been tested in pre-occupational contexts.

Meta-analytic evidence has demonstrated that several emotional skills including ERA facilitate higher work satisfaction and commitment with one’s work and, moreover, are predictive of higher well-being among teacher students [8,11,28]. However, the empirical evidence linking emotion regulation and pre-service teachers’ occupational commitment is quite scarce. Earlier studies have provided data on the association between overall EI and pre-service teachers’ occupational commitment, masking the particular role of the ability of managing one’s own and others’ emotions. For instance, a recent study testing the association between EI and commitment did not provide specific data on the role of emotion regulation dimensions as potential predictors of this dimension [9]. Although a previous work revealed a positive association between self-focused emotion regulation and commitment, unfortunately this study was conducted using a self-report instrument which does not account for the actual ability to manage one’s own emotions [24]. Therefore, it is necessary to generalize previous findings with self-report tests by using performance-based EI tests to provide evidence on the relationships between emotion regulation abilities and commitment.

Considering the work conducted by Hong [2], the capacity to manage emotions in one’s life or to manage others’ emotions may explain, to some extent, why beginning teachers who experience difficulties in dealing with emotions are more likely to display negative attitudes toward their jobs, which, eventually, affects their involvement and their withdrawal intentions. While researchers are beginning to recognize the role that emotion regulation may play in predicting well-being and performance in academic settings, the underlying mechanisms through which emotion regulation abilities may facilitate occupational commitment remain unclear. Considering the relevance of emotions in beginning teachers’ lives and in their academic motivation, adding to evidence regarding the effects of pre-service teachers’ emotion regulation ability on their engagement and their attitudes toward their career seems critical [29,30].

### 2.3. The Mediating Role of Study Engagement

The JD-R theory posits work engagement as a central topic of interest given its associations with a variety of personal, attitudinal, and motivational criteria [31,32]. This construct is gradually becoming a relevant dimension in pre-occupational settings given its positive relations with increased well-being, satisfaction, and commitment [13,33]. Study engagement is understood as an enduring, positive, fulfilling state of mind that is characterized by vigor, dedication, and absorption [34]. First, vigor refers to high levels of energy and mental resilience while studying. Vigorous students show willingness to invest effort in their study activities and show persistence in the face of difficulties. Second, dedication refers to being strongly involved in one’s studies with dedicated students showing a sense of inspiration from their studies as well as significance and enthusiasm. Finally, absorption refers to a state in which individuals are fully concentrated on their tasks and they feel time passes quickly. Although the three above-mentioned dimensions of engagement were originally distinguished, earlier studies have shown that vigor and dedication constitute the core dimensions of engagement [35,36]. Thus, in this study we measured these two dimensions.

Among the individual predictors of study engagement, the JD-R theory proposes that personal resources such as self-efficacy, psychological capital, and EI are antecedents of this construct through a motivational process [13,33,37,38]. Further, study engagement has been found as a mediator in the relationship between personal resources (i.e., psychological capital and self-efficacy) and outcomes such as academic performance or commitment [13,37]. According to JD-R theory applied to the teaching context, personal resources including emotion regulation would not only associate with increased levels of engagement but would be beneficial in their own right as they would lead to more positive work attitudes [39,40].

There are both theoretical and empirical reasons to expect pre-service teachers’ emotion regulation abilities to be associated with occupational commitment through study engagement. First, individuals with a more developed ability to regulate emotions would be more prone to make the most of their positive emotions regarding their tasks and activities and, hence, would show high levels of study engagement [30]. In this vein, a study with in-service teachers has found other-focused emotion regulation to be positively associated with work engagement [41], whereas a recent study with secondary school teachers has revealed ERA to be positively associated with work engagement [40]. The few available studies on EI and study engagement share the limitation that they have examined EI as a whole rather than providing data on dimensions including emotion regulation [33,42]. One exception is a study with trainees showing that self- and other-focused emotion regulation strategies were predictive of energy levels and learning behaviors on a weekly basis [27]. Second, personal resources such as ERA would provide individuals with a greater sense of control of their environment as well as a greater capacity to adjust to affective events that may impair their study-related motivation and their attitudes toward teaching career [2]. Third, ERA may help individuals to perceive a greater availability of resources in educational settings so emotionally savvy students may deploy more effort in their learning-related behaviors through increasing social resources such as seeking support from their supervisor [27].

According to the aforementioned studies, emotion-regulation abilities may help individuals to overcome study-related difficulties and to develop a greater sense of control of their emotions, which may influence their study engagement (i.e., vigor and dedication) and occupational commitment in a positive way. However, to date, no study has examined the associations among self- and other-focused emotion regulation, engagement, and occupational commitment among pre-service teachers.

Based upon previous theoretical and empirical evidence, the study hypotheses are:

**Hypothesis** **1a.** *Self-focused ERA is positively associated with occupational commitment.*

**Hypothesis** **1b.** *Other-focused ERA is positively associated with occupational commitment.*

**Hypothesis** **2a.** *Study engagement (study vigor and dedication) mediates the positive relationship between self-focused ERA and occupational commitment.*

**Hypothesis** **2b.** *Study engagement (study vigor and dedication) mediates the positive relationship between other-focused ERA and occupational commitment.*

## 3. Materials and Methods

### 3.1. Participants and Procedure

Participants were 249 students (65.5% female; M_age_ = 27 years) of a master’s degree in Teacher Training for Secondary Education at the University of Málaga. The participants were enrolled in this postgraduate training program comprising seventeen specialties including foreign languages (14.86%), counseling (17.67%), social sciences (11.24%), and technology (6.8%). Other specialties included mathematics (5.6%), physical education (5.6%), and economy (5.6%). Nineteen students did not report their specialty. Participants had no practical experience of teaching at the time of the study. Regarding their educational level, the majority (96.39%) of participants held either a five-year or a four-year degree, nine students held another master’s degree, and one participant held a doctorate.

Students were invited to participate in a study on “personal and contextual factors regarding teacher motivation” during their attendance at workshops. All potential participants were informed in depth about the data collection procedure. Specifically, they were given copies of a brief description of the research that stated its voluntary, individual, and confidential nature. They provided their informed consent as a requirement prior to filling in the questionnaires. In sum, this study was carried out in accordance with national and international ethical considerations and was approved by the ethics committee of the University of Málaga (66-2018-H).

### 3.2. Measures

Self- and other-focused emotion regulation abilities were measured using the emotion regulation subscale of the Mayer‒Salovey‒Caruso Emotional Intelligence Test (MSCEIT Version 2.0; [25]). In particular, the emotion regulation section includes 29 items and two tasks assessing the use of strategies for regulating emotions that are effective in handling one’s own emotions (with 20 items) and those of others (with 9 items). First, participants respond to the emotion management task in which they are asked to rate each item of a list of five scenarios with four actions or reactions in terms of its effectiveness for the proposed goal (e.g., maintaining an emotion). Second, participants are required to respond to the emotional relationships task which is composed of three scenarios with three responses each. The effectiveness of different actions aiming at achieving a particular other-focused emotional outcome is rated. The expert consensus method, which is found to be highly correlated with ratings derived from a general consensus sample, was considered to calculate the usefulness of the self- and other-focused actions [25]. Scores are calculated and rescaled as an individual’s deviation from the mean of the normative sample (mean = 100 and standard deviation = 15).

Internal consistencies of 0.55–0.88 have been reported for the different subtests [43]. We used a well-validated Spanish version [44]. In this study, split-half reliability was 0.76 for overall ERA. This is the preferred indicator for indicating reliability values for different branches with a variety of tasks. Regarding the specific tasks for one’s own and others’ emotions, Cronbach’s alpha is the appropriate measure for indicating reliability when intra-test homogeneity is found. For the emotion management subscale, Cronbach’s α was 0.60, whereas for the emotional relationship task it was 0.56.

Study vigor and dedication were evaluated with the Utrecht Work Engagement Scale adapted for students (UWES-S) [45]. This 15-item measure uses a seven-point Likert scale ranging from 1 (never) to 7 (always). Although it is comprised of three subscales evaluating vigor, dedication, and absorption with five items each, in line with previous research we measured vigor and dedication as the core dimensions of study engagement [35]. Sample items include “When studying I feel strong and vigorous” (vigor), and “I find my studies to be full of meaning and purpose” (dedication). For each dimension, items were summed and divided by the number of items of each subscale so that higher scores indicated greater levels of vigor and dedication. The Spanish adaptation was used [46]. In this study, Cronbach’s alpha was 0.85 for vigor and 0.89 for dedication.

Occupational commitment was assessed with a self-report instrument including six items assessing the level of attachment to the teaching career [12,17]. Participants are requested to respond using a Likert scale ranging from (1) “Disagree strongly” to (9) “Agree strongly” (e.g., “If I could do it all over again, I would not choose to work in the teaching profession,” reversed item). Scores were obtained by summing the responses and dividing them by the number of items so that higher scores indicated greater levels of occupational commitment. This instrument has shown adequate reliability in prior research involving pre-service teachers [12]. The Spanish version of the instrument has shown good psychometric properties with samples of pre-service teachers [9]. In this study, Cronbach’s alpha was 0.79.

Earlier research has reported age and gender differences in teacher retention indicators [5]. On this basis, the variables age and gender (0 = male, 1 = female) were included as covariates in the main analyses.

### 3.3. Analytic Plan

First, descriptive statistics (i.e., means, standard deviations, and internal consistency coefficients) were calculated for the main study variables. Second, correlation analyses were conducted to gain information on the associations amongst the main variables. Third, regarding the study hypotheses in relation to the proposed model (see Figure 1), the SPSS macro PROCESS (Model 4) was used [47]. Two independent multiple mediation models were estimated using bootstrapping techniques with 5000 resamples and a 95% bias-corrected confidence interval to obtain parameter estimates for both the total effect and the indirect effect models. Aiming to test the proposed conceptual model (see Figure 1), either self- or other-focused ERA was included as the independent variable (IV_1_ and IV_2_) in each model, whereas occupational commitment was entered as the dependent variable (DV) and study vigor and dedication were tested as the mediator variables (M_1_ and M_2_).

## 4. Results

### 4.1. Descriptive Results: Confirmatory Factor Analyses and Bivariate Correlations

First, confirmatory factor analysis was conducted to test the reliability and the validity of study vigor, study dedication, and occupational commitment. The main results are shown in Table 1. Two items from the occupational commitment scale were excluded as their factor loadings were low and the average variance extracted (AVE) of the measure was below the threshold of 0.50. After excluding these items, error terms of items from the variables study vigor, study dedication, and occupational commitment were specified to correlate considering the suggestion from the modification indices. The factor loadings of all items were around or above 0.50. The final measurement model including the three dimensions (i.e., study vigor, study dedication, and occupational commitment) was satisfactory (X^2^ = 124.13, df = 68, CMIN/df = 1.83, P-close = 0.21; RMSEA = 0.06; SRMR = 0.06; CFI = 0.97; TLI = 0.96). In sum, the results yielded adequate validity and reliability.

Second, regarding means, scores reported were medium in self- and other-focused ERA and also in vigor, whereas the scores in dedication and occupational commitment were high. Third, bivariate correlations for the study variables were calculated. Table 2 shows the bivariate correlations for the study variables. As shown, self-focused and other-focused ERA were positively associated with study vigor, study dedication, and occupational commitment. Similarly, study vigor and dedication were positively related to occupational commitment.

### 4.2. Mediator Model with Self-Focused ERA as Predictor

A multiple mediator model regarding the relationship between self-focused ERA and occupational commitment through study vigor and dedication was tested. Main results are presented in Table 3. With respect to study vigor, results showed that the coefficients of paths *a*_1_ and *b*_1_ were significant, illustrating a positive association of self-focused ERA on vigor (*b* = 0.02, *p* < 0.001) and a positive relation between vigor and occupational commitment (*b* = 0.41, *p* < 0.01). The residual direct effect of self-focused ERA on occupational commitment became insignificant when vigor was included in the model (path c’; *b* = 0.01, *p* = 0.24). Moreover, a significant indirect effect was found in the relationship between self-focused ERA and occupational commitment through vigor (path *a*_1_*b*_1_; *b* = 0.008; BCa 95% CI = 0.002 to 0.016). Thus, the results supported that vigor fully mediated the relationship between self-focused ERA and occupational commitment.

Regarding the mediator effect of study dedication, results showed a positive association between self-focused ERA and dedication (path *a*_2_; *b* = 0.02, *p* < 0.001). However, there was a non-significant association between dedication and occupational commitment (path *b*_2_; *b* = 0.07, *p* = 0.56). In sum, the results supported a significant indirect effect of self-focused ERA on occupational commitment through study vigor, whereas dedication was not found as a significant mediator in this relationship.

### 4.3. Mediator Model with Other-Focused ERA as Predictor

Mediator analyses were conducted for other-focused ERA as predictor. With regard to vigor, results showed a non-significant coefficient for the path *a*_1_ regarding the association between self-focused ERA and vigor (*b* = 0.01, *SE* = 0.01, *t* = 1.77, *p* = 0.08, 95% CI = −0.001, 0.020). Moreover, the point estimate regarding the indirect effect through vigor (path *a*_1_*b*_1_) was not significant (*b* = 0.004, *SE* = 0.003, 95% CI = 0.000, 0.010). Regarding dedication, results showed a significant coefficient for the path *a*_2_ on the association between self-focused ERA and dedication (*b* = 0.02, *SE* = 0.01, *t* = 2.80, *p* < 0.01, 95% CI = 0.004, 0.025), indicating a positive association between the two variables. However, the coefficient for the path *b*_2_ was not significant (*b* = 0.07, *SE* = 0.12, *t* = 0.56, *p* = 0.57, 95% CI = −0.017, 0.030). Similar non-significant results were found for the mediator variable dedication (path *a*_2_*b*_2_; *b* = 0.001, *SE* = 0.002, 95% CI = −0.003, 0.007). The total effect (path *c*) of other-focused ERA on occupational commitment was significant (*b* = 0.02, *SE* = 0.01, *t* = 2.10, *p* < 0.05, 95% CI = 0.001, 0.034), whereas the direct effect (path *c*′) was non-significant (*b* = 0.01, *SE* = 0.01, *t* = 1.54, *p* = 0.12, 95% CI = −0.004, 0.030). In sum, results indicated that neither vigor nor dedication were significant mediators in the association between other-focused ERA and occupational commitment.

**Figure 2:**
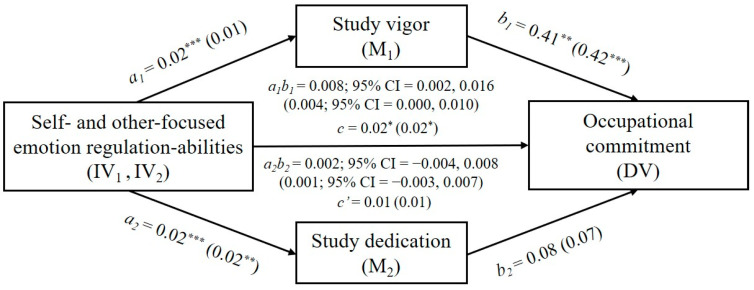
Graphical representation of the final mediator model. Note. Results for other-focused ERA as predictor are in parentheses. * *p* < 0.05; ** *p* < 0.01; *** *p* < 0.001.

Figure 2 illustrates the data regarding the proposed mediator model with self- and other-focused ERA as predictors.

## 5. Discussion

The current study extended previous studies on EI and pre-service teachers’ engagement and commitment by testing the relationship between study vigor and dedication in the relationship between self- and other-focused emotion regulation abilities and occupational commitment. This work goes beyond earlier research using overall EI scores or self-reported instruments and provides preliminary findings suggesting that pre-service teachers with a higher ability to manage their own emotions are more likely to experience high levels of mental resilience and energy while working, thereby showing a higher willingness to invest efforts in their tasks and activities related to teaching and, in turn, a higher commitment to their teaching career. Results on hypotheses 1a and 1b were consistent with previous studies showing pre-service teachers’ self-focused emotion regulation to be associated with a higher commitment to teaching using self-report measures [24]. However, results provided mixed support for hypothesis 2a and did not show significance for hypothesis 2b.

One potential explanation for the mixed finding regarding hypothesis 2a, that is, self-focused ERA was a positive predictor of occupational commitment through study vigor (but not dedication), could be due to the nature of study engagement. Results revealed that self-focused ERA may help pre-service teachers to deal with hindrance demands (i.e., study workload) that may undermine their energy levels and could diminish their persistence in the face of obstacles and difficulties. Moreover, they suggested self-focused emotion regulation as a personal resource increasing energy and mental energy among future teachers so they feel more capable of overcoming future challenges that they may encounter during their traineeship and explaining their higher commitment to a teaching career. While vigor is a study engagement dimension linked to levels of energy, dedication is a dimension rather associated with levels of significance, challenge, and inspiration regarding academic tasks. It is plausible, then, that there are other variables that were not measured and that may associate with study dedication to a greater extent than self-focused ERA. For instance, academic resources such as tasks variety or teachers’ provision of autonomy support are critical contextual factors that may account for a significant amount of variance in study dedication [34]. Some initial evidence on study engagement dimensions is found. Therefore, further studies are needed to test the possible association between self- and other-focused emotion regulation abilities and study dedication.

An explanation for the non-significant findings regarding hypothesis 2b could be given regarding recent works on the efficacy of specific intrapersonal or interpersonal facets of EI. A study with undergraduate students showed a positive association between self-focused regulation and energy levels on a weekly basis, whereas other-focused emotion regulation was not associated with energy levels [27]. The pre-service teacher sample had no practical experience of teaching at the time of data collection and this fact may explain why other-focused ERA was not associated with occupational commitment through study vigor or dedication. It is reasonable that other-focused ERA may be predictive of occupational commitment in the context of teaching practice in which there are a number of interpersonal relationships. For instance, other-focused ERA could help trainees to be more skilled at shaping their students’ emotions (i.e., to improve classroom management or to increase teacher-student connectedness) or it may help pre-service teachers receive greater support from their colleagues and principals. These processes relying on their ability to manage others’ emotions could eventually lead them to feel more engaged and committed to teaching [23,26,27]. In sum, our preliminary findings indicated that the ability to manage one’s own emotions may be effective for maintaining well-being [24,27].

### 5.1. Theoretical and Practical Implications

With respect to the theoretical implications of these findings, this study may provide a greater insight into the predictive role of personal resources involving emotion regulation abilities relating to pre-service teachers’ engagement and commitment with their career. The applicability of these results may help researchers to devote efforts to addressing the role of individual resources and study motivation in the pre-occupational stages so future workers feel more prepared and committed to their careers [11,13]. This study reported insightful preliminary findings suggesting independent processes of the utility of ERA which may indicate that pre-service teachers could benefit from their self-focused abilities to maintain energy levels in the face of study demands and to display more positive attitudes toward teaching practice and development as secondary school teachers [4,20]. Given the severe personal and occupational consequences of stress, burnout, and presenteeism, studies focusing on the role of teachers’ emotion regulation abilities would contribute to this field [14,48]. One of the theoretical contributions of our study relates to the finding that a key emotional ability accounting for an individual’s capacity to manage their own emotions associates with greater vigor and occupational commitment. Taken together, these results may contribute to the field of environmental research and public health as they may suggest the development of a future research line testing the potential contribution of emotion regulation abilities to adaptive coping with contextual factors (i.e., demanding and/or challenging situations at school) accounting for levels of motivation and commitment in educational contexts [1,39]. Finally, these mixed findings point to the need to conduct studies on the effects of self- and other-focused EI dimensions and analyzing their specific effects on well-being and commitment [49]. These studies could elucidate whether other-focused emotion regulation abilities are helpful to improve teachers’ classroom management and their connectedness with students rather than their own energy and well-being. In sum, these preliminary findings highlight the need for future studies applying the JD-R theory in pre-occupational stages to delve into the effects of self- and other-focused emotion regulation abilities on motivation and attitudinal indicators.

Regarding practical implications, future research is advised to promote self-focused emotion regulation strategies among pre-service teachers during their training as a worthwhile strategy to enhance levels of vigor and commitment. According to prior research on EI training with pre-service teachers, university teachers working with pre-service secondary education teachers may focus on the implementation of programs including self-focused regulation strategies aiming at reducing stress related to academic tasks and demanding situations at university [7,50]. Further, these results point out the predictive role of self-focused ERA on motivation and commitment. Thus, future training programs on emotion regulation during traineeships and teaching practice may help future teachers to deal with stress in a more efficient way [4]. It has been argued that emotional abilities may be critical to reduce teacher stress and so these findings may be helpful in the design of multi-faceted positive organizational psychology interventions in an effort to combat teacher burnout and to facilitate motivation and commitment [1].

Findings from this study indicate that specific training on how to maintain desirable states when dealing with study-related factors should be included in the postgraduate training for pre-service teachers. For instance, training on the use of adaptive self-focused emotion regulation strategies (e.g., focusing on planning or reappraisal) may help individuals to improve their management of stress-eliciting situations (e.g., study workload) and reduce the likelihood of subsequent emotional reactions (e.g., frustration, exhaustion) that may erode their commitment and involvement. This training may be particularly critical for pre-service teachers facing interpersonal demands in teaching such as classroom management issues or students’ lack of motivation [2,3,4]. This approach at the pre-service stage appears to be promising so that beginning teachers perform better in stressful contexts such as secondary education [11,24].

### 5.2. Limitations and Future Research Avenues

Some limitations of this work should be considered. First, future research should profitably address longitudinal designs to test whether pre-service teachers who are more skilled regarding self-focused emotion regulation abilities are more likely to stay vigorous and committed to the teaching profession during their first years of experience. In light of longitudinal evidence revealing the predictive effect of specific EI dimensions on subjective well-being and burnout among future health professionals, this line seems worthwhile [51]. Similarly, a limitation of the study is associated to the lack of practical teaching experience of the sample. This fact might explain the need to exclude two items of the occupational commitment scale. One potential explanation is that participants may not sufficiently have educational practices at school and that this variable may have influenced their responses to the occupational commitment scale. Accordingly, future research is needed to replicate current findings with the complete scale in more experienced novel teachers to examine the generalizability of our results [12]. For instance, it could be tested whether scores in this occupational commitment scale would be different between pre-service teachers with less vs. more experience regarding practical traineeship and how it might show evidence for the differential influence on studied variables [9]. Future studies should profitably test whether other-focused emotion regulation skills are predictive of their vigor levels and their occupational commitment in the face of interpersonal demands such as classroom management [20]. Considering the high attrition rates among beginning teachers who are dealing with sources of stress including students’ misbehavior, these issues merit further attention [4].

A second limitation is related to the relatively low internal consistency of the dimensions regarding ERA. Although the values were found to be in the interval proposed by the authors [43], future studies are expected to test the examined relationships including complementary measures to test emotion regulation abilities in relation to teaching-specific situations. For instance, using situational judgment tests adapted to the challenges pre-service teachers may face in their professional development may beneficially contribute to evidence linking the implementation of these abilities with lower exhaustion and greater satisfaction [52]. Moreover, research is needed to test the underlying mechanisms through which self-focused emotion regulation leads to higher study vigor and increased commitment. In this regard, it should be acknowledged that the amount of variance in occupational commitment explained by self-focused ERA and vigor was relatively low. Although performance-based EI tests show a lower predictive effect on work-related criteria than self-report EI instruments, it is plausible that there were other relevant variables that we were unable to include in the proposed model and that would show a predictive role on occupational commitment (e.g., self-efficacy, [11]). Therefore, it would be valuable in future comprehensive works to test the joint contribution of personal resources (e.g., emotion regulation abilities, resilience, or self-efficacy beliefs) and contextual factors (e.g., professional support from colleagues or perceived autonomy support) to increased vigor and commitment [2,52,53]. Such an approach would beneficially advance knowledge in the fields of environmental research and public health as it might point out to the most relevant conditions and resources facilitating well-being among novice teachers [1,2]. Finally, positive outcomes of engagement such as active learning and creativity could be examined as desirable indicators among future teachers [27,54]. Future studies conducted with samples of pre-service teachers during their practical experiences and exploring the explanatory role of study engagement dimensions may advance knowledge on the utility of self- and other-focused emotion regulation to benefit well-being and career adaptability [4,27].

## 6. Conclusions

Limitations notwithstanding, this research is the first attempt to test self- and other-focused ERA and study engagement as predictors of pre-service teachers’ occupational commitment. Results showed findings suggesting the differential role emotion regulation abilities may exert for explaining energy levels and an increased attachment to working in teaching. Beyond efforts directed at reducing stress and exhaustion among beginning teachers, these findings suggest the potential value of incorporating development of emotional abilities to facilitate desirable positive outcomes in educational settings. Thus, strategies to improve pre-service teachers’ self-focused emotion regulation abilities and study vigor are expected to contribute to boosting positive attitudes toward teaching so a desirable outcome such as teacher commitment is facilitated.

## Figures and Tables

**Table 1:** Descriptive statistics of constructs derived from the questionnaire survey.

	Mean	SD	Range	Min.	Max.	α	CR	AVE
1. Self-focused ERA	101.56	12.81	71.70	47.83	119.54	0.60	-	-
2. Other-focused ERA	105.38	11.98	61.56	58.65	120.21	0.56	-	-
3. Study vigor	4.30	1.05	5.40	0.60	6.00	0.85	0.84	0.52
4. Study dedication	5.01	1.03	5.60	0.40	6.00	0.89	0.90	0.64
5. Occupational commitment	7.59	1.59	7.75	1.25	9.00	0.79	0.80	0.51

Note. Two items from the occupational commitment scale were excluded as they showed low values regarding their factor loadings. ERA = emotion regulation ability; SD = standard deviation; Min. = minimum; Max. = maximum; α = Cronbach’s alpha; CR = composite reliability; AVE = average variance extracted.

**Table 2:** Descriptive statistics and correlations among study variables.

	1	2	3	4	5
1. Self-focused ERA	-				
2. Other-focused ERA	0.49 **	-			
3. Study vigor	0.28 **	0.15 *	-		
4. Study dedication	0.29 **	0.21 **	0.60 **	-	
5. Occupational commitment	0.15 *	0.13 *	0.30 **	0.21 **	-

Note. *N* = 249. * *p* < 0.05; ** *p* < 0.01. ERA = emotion regulation ability.

**Table 3:** Indirect effects of self-focused ERA on occupational commitment through study vigor and dedication.

	Total Effect Model				Indirect Effect Model			
Path	B	SE	*t*	95% Bias-Corrected CI	B	SE	*t*	BCa 95% CI
Age ^a^	0.02	0.02	1.04	(−0.02, 0.05)	0.01	0.02	0.34	(−0.03, 0.04)
Gender ^a^	−0.13	0.21	−0.63	(−0.54, 0.28)	−0.30	0.20	−1.46	(−0.70, 0.10)
Self-focused ERA-vigor (*a*_1_)	0.02	0.01	3.79 ***	(0.01, 0.03)				
Self-focused ERA-dedication (*a*_2_*)*	0.02	0.01	4.17 ***	(0.01, 0.03)				
Vigor-occupational commitment (*b*_1_)	0.41	0.12	3.48 **	(0.18, 0.64)				
Dedication-occupational commitment (*b*_2_)	0.07	0.12	0.59	(−0.17, 0.31)				
Self-focused ERA-occupational commitment (*c*)	0.02	0.01	2.33 *	(0.003, 0.034)				
Self-focused ERA-occupational commitment (*c*′)	0.01	0.01	1.54	(−0.00, 0.03)				
Self-focused ERA-vigor-occupational commitment (*a*_1_*b*_1_)					0.008	0.004		(0.002, 0.016)
Self-focused ERA-dedication-occupational commitment (*a*_2_*b*_2_)					0.001	0.003		(−0.004, 0.008)
R^2^	0.03				0.11			
F (df)	2.43 * (3, 245)				5.79 *** (5, 243)			

Note. *N* = 249. ERA = emotion regulation ability. Letters *a*, *b*, *c*, and *c*′ indicate unstandardized regression coefficients: *a*_1_ = direct association between self-focused ERA and vigor; *a*_2_ = direct association between self-focused ERA and dedication; *b*_1_ = direct association between study vigor and occupational commitment; *b*_2_ = direct association between study dedication and occupational commitment; *c* = total effect between self-focused ERA and occupational commitment (not accounting for study vigor nor study dedication); *c*′ = direct effect between self-focused ERA and occupational commitment (accounting for study vigor and dedication). Letters *ab* indicate unstandardized coefficients: *a*_1_*b*_1_ = indirect effect between self-focused ERA and occupational commitment through study vigor; *a*_2_*b*_2_ = indirect effect between self-focused ERA and occupational commitment through study dedication. BCa 95% CI = bias-corrected and accelerated 95% confidence interval with 5000 resamples. ^a^ Age and gender were included as covariates. * *p* < 0.05; ** *p* < 0.01; *** *p* < 0.001.

## Data Availability

The datasets presented in this article are not readily available because the dataset has been generated regarding a funded Research Project by Junta de Andalucia/FEDER funds (UMA18- FEDERJA-147). Requests to access the datasets should be directed to NE, nextremera@uma.es.
